# Brown spiders (Loxosceles) are taking hold in Pernambuco, Brazil: a case series, 2018-2022

**DOI:** 10.1590/S2237-96222024v33e2023568.en

**Published:** 2024-01-15

**Authors:** Maria Lucineide Porto Amorim, Diógenes Gayo de Oliveira Simão, João Paulo Vieira e Silva de Albuquerque, Beatriz Maria Tenório Ramos, Gustavo José Lopes do Nascimento, Maria Júlia Gonçalves de Mello

**Affiliations:** 1 Centro de Informação e Assistência Toxicológica de Pernambuco, Recife, PE, Brazil; 2 Instituto de Medicina Integral Prof. Fernando Figueira, Programa Institucional de Bolsas de Iniciação Científica, Recife, PE, Brazil; 3 Faculdade Pernambucana de Saúde, Curso de Medicina, Recife, PE, Brazil; 4 Instituto de Medicina Integral Prof. Fernando Figueira, Departamento de Pós-Graduação Stricto Sensu, Recife, PE, Brazil

**Keywords:** Spiders, Poisoning, Epidemiology, Public Health Surveillance, Health Surveillance, Case Reports, Arañas, Intoxicación, Epidemiología, Vigilancia en Salud Pública, Vigilancia Sanitaria, Informes de Casos, Aranhas, Envenenamento, Epidemiologia, Vigilância em Saúde Pública, Vigilância Sanitária, Relatos de Casos

## Abstract

**Objective:**

To describe accidents involving brown spider (genus Loxosceles) bites notified by the Pernambuco Poison Information and Care Center (CIATox-PE), Brazil, from January 2018 to December 2022.

**Methods:**

This was a case series study of brown spider bites notified by the CIATox-PE.

**Results:**

The study included 22 cases with median age of 35 years, the majority being female (13); the cases occurred in rural and urban areas (12 *versus* 10), at night (10); Petrolina was the municipality with the highest number of notifications (6); spider bites occurred mainly in the lower (11) and upper (9) limbs, almost exclusively inside households (21); specific serum therapy was not indicated for 8 cases because the time for its effectiveness had already elapsed.

**Conclusion:**

Loxoscelism cases occurred more frequently in females, in both rural and urban areas and mainly at home, with delays in seeking medical care.

## Study contributions

**Main results:**

The 22 cases described represent an alert for the presence of a venomous species not reported in previous studies in Pernambuco. The severity of cases may be associated with delays in seeking medical care, especially due to the population's lack of information.

**Implications for services:**

Identify the presence of brown spiders in households in the region and the potential severity of poisonings. Alert health professionals to recognize the clinical picture with a view to appropriate early treatment.

**Perspectives:**

Develop research on poisoning by venomous animals and educational actions alerting the population to prevent these accidents and updating health professionals on care.

## RESUMEN

**Objetivo:**

Describir accidentes causados ​​por arañas pardas (género Loxosceles) notificados por el Centro de Información de Asistencia Toxicológica de Pernambuco (CIATox-PE), Brasil, de enero de 2018 a diciembre de 2022.

**Métodos:**

Reporte de 22 casos notificados de CIATox-PE.

**Resultados:**

Casos con media de idade de 35 años, predominio femenino (13); los casos ocurrieron en área rural/urbana (12 versus 10), por la noche (10); Petrolina fue el municipio con más notificaciones (6); las picaduras fueron principalmente en los miembros inferiores (11) y superiores (9), casi exclusivamente en el interior de las viviendas (21); en 8 de los casos no se indicó sueroterapia específica por haber superado el tiempo de efecto.

**Conclusión:**

Los casos de loxoscelismo ocurrieron con mayor frecuencia en el sexo femenino, en áreas rurales y urbanas, y principalmente en el hogar, con demoras en la búsqueda de atención médica.

## RESUMO

**Objetivo:**

Descrever os acidentes por picada de aranhas-marrons (gênero Loxosceles), notificados no Centro de Informação de Assistência Toxicológica de Pernambuco (CIATox-PE), Brasil, no período de 2018 a 2022.

**Métodos:**

Estudo de série, sobre casos notificados no CIATox-PE.

**Resultados:**

Foram incluídos 22 casos com mediana de idade de 35 anos e houve predomínio do sexo feminino (13); os casos ocorreram nas zonas rural e urbana (12 *versus* 10), durante a noite (10), e Petrolina foi o município com mais notificações (6); as picadas ocorreram, principalmente, nos membros inferiores (11) e superiores (9), quase exclusivamente dentro das residências (21); para 8 acidentados, não se prescreveu soroterapia específica por terem ultrapassado o tempo de efetividade.

**Conclusão:**

Os casos de loxoscelismo ocorreram com maior frequência no sexo feminino, nas zonas rural e urbana indiferentemente, quase todos foram intradomiciliares e houve demora na procura por atendimento médico.

## INTRODUCTION 

Spiders are one of the groups of venomous species that most cause poisoning in Brazil, with great variation in frequency between the country’s five macro-regions and time of year.^1-5^ Brown spiders are arachnids comprising different species of the genus Loxosceles that can cause spider bite injuries.^6,7^ In the Northeast region of Brazil, especially in the state of Pernambuco, an increase in notifications of loxoscelism cases on the Brazilian System for Recording Poison Information and Care Center Poisoning Data (DATATOX) was seen between 2018 and 2022.^8^

Loxosceles bites give rise to a characteristic syndrome, given the dermonecrotic and hemolytic action of the venom, forming an ulcer at the site of the bite, this being the most common clinical finding of cutaneous loxoscelism. The generalized condition resulting from hemolysis involves acute anemia, jaundice and hemoglobinuria,^2,3,9,10^ the prognosis of which, generally favorable, is associated with a low case fatality ratio, but depends on the time elapsed between being bitten and receiving medical care.^2,3,10-13^

Recognized by the World Health Organization (WHO), the Pan American Health Organization (PAHO-WHO) and the Brazilian Ministry of Health, with effect from 2010 venomous animal poisoning has been a health condition requiring compulsory notification.^1,14-18^ However, underreporting and little epidemiological importance given to accidents involving venomous animals means that loxoscelism continues to be part of the group of neglected diseases.^18,19,21,24^

Due to lack of information, people affected by Loxosceles do not attach importance to the initial bite injury and delay seeking health services, thus hindering timely diagnosis and appropriate therapeutic actions.^6,11,20,24^

The objective of this study was to describe Loxosceles accidents notified by the Pernambuco Poison Information and Care Center (*Centro de Informação e Assistência Toxicológica de Pernambuco* - CIATox-PE), between 2018 and 2022. 

## METHODS 

This study analyzed a series of cases notified on the DATATOX, the CIATox-PE case recording system.^8^ Pernambuco has approximately 9 million inhabitants, distributed over 98,067.877 km² and 184 municipalities, which in turn are grouped into 12 Regional Health Management Districts (*Gerências Regionais de Saúde* - GERES).^25^

The database chosen for this study was the DATATOX, a computerized system for recording, monitoring, storing, processing and retrieving data on cases of exposure to toxic agents, cared for by CIATox throughout Brazil. The study content and concepts, involving notification forms used by some CIATox, were based on the model of the American Association of Poison Control Centers (AAPCC) and the International Program on Chemical Safety (INTOX/IPCS). We also sought to verify other poisoning notification models that exist in Brazil, such as the Notifiable Health Conditions Information System (*Sistema de Informação de Agravos de Notificação* - SINAN) and the Health Surveillance Notification System (*Sistema de Notificações em Vigilância Sanitária* - e-Notivisa); however, DATATOX is an exclusive system for cases reported by the CIATox, while the SINAN covers a wide range of diseases and conditions subject to compulsory notification and consequently does not monitor the evolution of each notified case.^26^

The DATATOX system was accessed by the researchers remotely, on January 13, 2023. Loxosceles accidents from January 2018 to December 2022 notified on electronic medical records were included in the study, while cases with unconfirmed information were excluded.

The variables we studied are found on the case notification form:

- a) age (last birthday);
- b) sex (male; female);
- c) area in which exposure occurred (rural; urban);
- d) place of occurrence (household; external environment);
- e) municipality;
- f) date;
- g) time of day of exposure (morning; afternoon; night);
- h) bite topography (head; torso; limbs);
- i) time between bite and medical care (in hours);
- j) need for hospitalization (yes; no);
- k) poisoning treatment (specific serotherapy; corticosteroid; antimicrobial (antibiotics); debridement);
- l) length of hospital stay (in days); and
- m) outcome [cure; sequela; lost to follow-up (indivduals not located for case closure)].

Loxoscelism case severity classification (in three degrees: mild cutaneous; moderate cutaneous; severe cutaneous) was based on a Brazilian Ministry of Health manual.^2,3,14^ Stata 13.0 software was used for the descriptive analysis of the cases in absolute values. 

The study project and a Letter of Consent from the Pernambuco State Health Department were submitted to and approved by a Human Research Ethics Committee: Certificate of Submission for Ethical Appraisal (*Certificado de Apresentação para Apreciação* Ética - CAAE) No. 60909922.5.0000.5201. As this was a descriptive observational study, with data collection comprising medical information acquired in accordance with an institutional protocol, the study was exempt from needing Free and Informed Consent, given that the confidentiality of the research participants’ data was ensured.

## RESULTS

Of the 25 cases reported as loxoscelism, 22 were eligible; the remaining three were excluded due to lack of conclusive information about diagnosis. 

**Figure 1:**
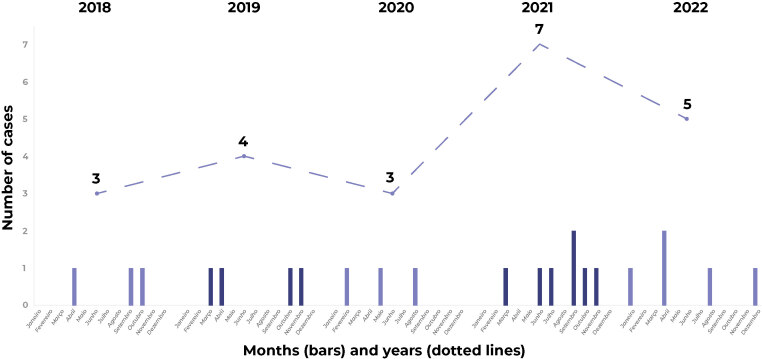
Distribution of DATATOX^a^ loxoscelism notifications, by month and year of occurrence, Pernambuco, Brazil, 2018-2022

The distribution of the 22 cases analyzed, according to the year and month of the accident, is shown in Figure 1: 3 cases in 2018; 4 in 2019; 3 in 2020; 7 in 2021; and 5 in 2022. Median age was 35 years, with an interquartile range of 20 to 45; there was a predominance of females, accounting for 13 cases. 

a) DATATOX = Brazilian System for Recording Poison Information and Care Center Poisoning Data (*Sistema Brasileiro de Registro de Intoxicações dos Centros de Informação e Assistência Toxicológica*).

**Figure 2:**
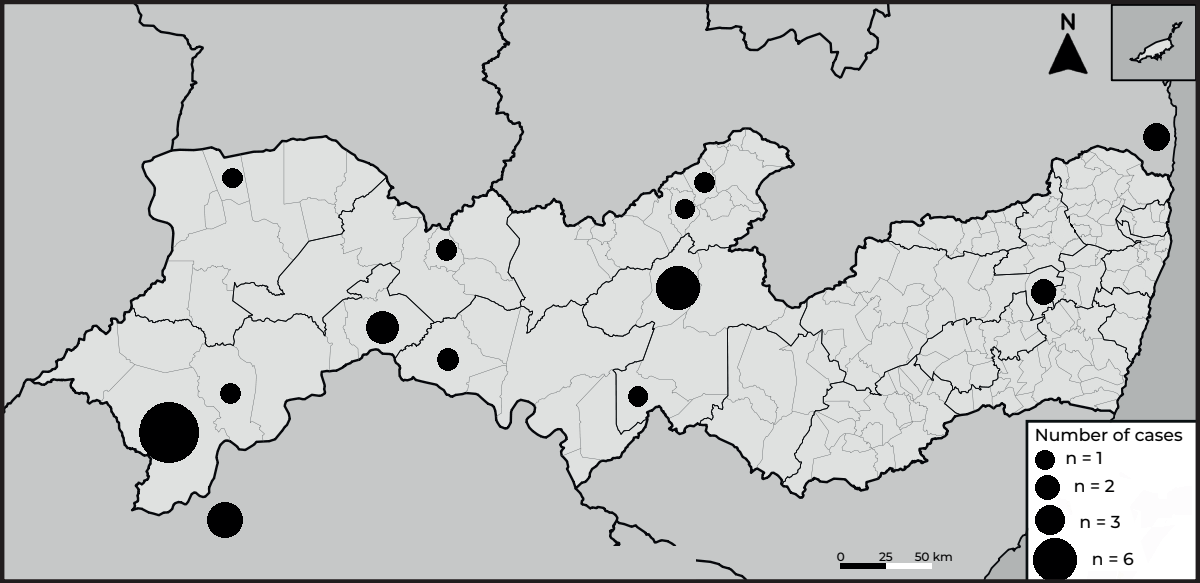
Geographic distribution of loxoscelism cases notified by the CIATox-PE,^a^ Pernambuco, Brazil, 2018-2022

Legend: PE = Pernambuco; BA = Bahia; PB = Paraíba. a) CIATox-PE = Pernambuco Poison Information and Care Center (*Centro de Informação de Assistência Toxicológica de Pernambuco*). Notes: The black circles correspond to the number of cases per municipality, scale 1:2,500,000, with map orientation (north) corresponding to the convention. Circles outside Pernambuco correspond to two cases exposed in Juazeiro (Bahia) and Conde (Paraíba).

The geographic distribution, according to the state’s municipalities, is shown in Figure 2: Petrolina, with 6 cases; Custódia, with 3; Juazeiro, Paulista, Cabrobó and Afogados da Ingazeira, each with 2 cases; Ipojuca, Lagoa Grande, Recife, Bélem de São Francisco, Guarulhos, Ipubi, Inajá and Limoeiro, each with 1 case. 

Epidemiological and clinical characteristics of the spider bites are summarized in Table 1. These accidents occurred in rural areas (12) and in urban areas (10), with greater occurrence at night (10). The bites occurred, almost exclusively, inside households (21). Bite topography was distributed almost equally between the lower (11) and upper (9) limbs. 

**Table 1:** Epidemiological and clinical characteristics of the 22 loxoscelism cases notified at the CIATox-PE,^a^ Pernambuco, Brazil, 2018-2022

Variables	Cases
**Epidemiological characteristics**	
**Sex**	
Male	9
Female	13
**Area in which exposure occurred**	
Urban	10
Rural	12
**Place of exposure**	
Usual residence	21
Outside/public environment	1
**Time of day of exposure**	
Morning	8
Afternoon	4
Night	10
**Clinical characteristics**	
**Classification of severity**	
Mild cutaneous	4
Moderate cutaneous	7
Severe cutaneous	10
Systemic	1
**Hospitalization**	
Yes	18
No	4
**Poisoning treatment**	
Specific serotherapy	11
Corticosteroid	14
Antimicrobial (antibiotics)	16
Debridement	7
**Outcome**	
Cure	19
Sequela	1
Lost to follow-up	2

a) CIATox-PE: Pernambuco Poison Information and Care Center (Centro de Informação de Assistência Toxicológica de Pernambuco).

Median time elapsed between being bitten and receiving medical care was 48 hours, with 12 of the individuals treated after 36 hours. Hospitalization occurred in 18 cases, with a median hospital stay of 5.5 days. Among the treatments carried out, serotherapy with anti-arachnid serum or anti-loxocelic serum was adopted in 11 cases, while corticosteroids were used in 14 cases and antimicrobials in 16. Due to having exceeded the effective time of anti-arachnid serum therapy, 8 individuals did not receive specific serotherapy. Wound debridement was performed in 7 cases.

The majority of cases were classified as moderate and severe (17). Reversible kidney injury occurred in one of the cases and the most frequent complication was wound infection, in 6 cases. Two individuals were lost to follow-up, and the majority (19) were cured; a single case, which presented pyoderma gangrenosum, required amputation of the lower left limb. 

## DISCUSSION

The epidemiological characteristics of the notified cases were similar to those of the cases described in the literature, differing only in terms of the greater frequency of moderate and severe clinical conditions, probably associated with taking more time to seek medical care. 

Adults were most affected, with no difference between the sexes. These results are in line with those of other research carried out in the southern region of Brazil for the period 2019-2021.^5,6,27^ The first notifications corresponded to the *Sertão* region of the state of Pernambuco, followed by occurrences in municipalities closer to the coast, suggesting geographic displacement of cases. Rural and urban areas had similar notifications in numbers, unlike the cases cited in the Ministry of Health manual for the years 2001-2019, for which the urban area was the main place of occurrence, perhaps due to the greater demographic density in rural areas in northeast Brazil when compared to rural areas the southern region of the country.^2,3,25^ As the Loxosceles spider usually inhabits domestic spaces, almost all accidents occurred in households, the expected habitat of these spiders, according to findings from other research carried out in southern Brazil for the period 2001-2019.^2,3,5,28^

Median time elapsed between the victim being bitten and receiving care was 48 hours, this being higher than that found in reviews of the literature and research on endemic areas in the state of Paraná and in Chile for the period 2021-2022, probably due to a lack of common knowledge about brown spiders, difficulty in accessing health services and initial injury not characteristic of brown spider bite.^6,14,27^ This time interval is worrying as it implies a prognosis for those affected: use of specific serum is indicated in moderate and severe cases, being more effective the sooner it is applied. The Ministry of Health recommends that it be administered within 36 hours.^3,10,14^ A considerable number of moderate and severe cases did not receive serotherapy because the time needed for its adequate use had already elapsed.^14^

Clinical classification as “severe cutaneous form” and “moderate cutaneous form” was probably more frequent due to the delay in seeking medical assistance, which led to the worsening of the condition, unlike cases that receive serum therapy in time.^6,26,29^ Most cases were hospitalized and were treated with antimicrobials, given the presence of signs of infection in the wound, requiring debridement. Cases reported as being of the “severe cutaneous form” had larger lesions, slower evolution and longer hospitalization. Lack of information among the population about the severity of accidents could possibly account for underreporting of mild cases, and could have increased the proportion of severe and moderate cases.

The majority of accident victims recovered without sequelae. However, one of them took 24 days to seek health care and developed pyoderma gangrenosum as a complication, requiring 60 days of hospitalization and amputation of their lower left limb. Excluding this case did not lead to an important change in the assessment of the time elapsed – considered high – before seeking health services.

Given the small number of cases in the sample, the results and hypotheses raised have limited applicability, and further research is recommended. The loxoscelism cases in Pernambuco presented epidemiological characteristics similar to those described for endemic regions. The delay in seeking health care should serve as a warning for health surveillance and health care services, so that the population and health professionals involved be better informed about the presence of the brown spider (Loxosceles), the possible complications arising from brown spider bites and the importance of prevention measures, aiming to reduce morbidity caused by them.

## References

[1] Konstantyner TCRO, Martins CB, Góis AFT, Castro BVC, Konstantyner T (2022). Trend in the incidence rates of accidents with venomous animals
in children and adolescents in Brazil (2007–2019). Rev Paul Pediatr.

[2] Fundação Nacional de ﻿Saúde (2001). Manual de diagnostico e tratamento de acidentes por animais
peçonhentos.

[3] Ministério da Saúde (BR) (2019). Guia de vigilância em saúde.

[4] Nunes MLC, Farias JACR, Anselmo DA, Anselmo MA, Andrade RFV (2022). Acidentes com animais peçonhentos no Brasil: uma revisão
integrativa. Arq Cien Saude UNIPA.

[5] Souza TC, Farias BES, Bernarde PS, Chiaravalotti F, Frade DDR, Brilhante AF (2022). Tendência temporal e perfil epidemiológico dos acidentes por
Animais Peçonhentos no Brasil, 2007-2019. Epidemiol Serv Saude.

[6] Benedet DP, Bertan FAB, Zorzan M, Tessaro D (2021). Epidemiologia do araneísmo por loxosceles e phoneutria no
município de Cruzeiro do Iguaçu, Paraná – Brasil. Rev Cienc Med Bio.

[7] Silva BT, Hernandes PR, Lopes PAC, Barros LM, Gomes TV, Rosa JS (2021). Doenças tropicais negligenciadas: uma análise dos acidentes por
animais peçonhentos (2010-2019). Rev Cientifica Integrada.

[8] Associação Brasileira de Centros de Informação e Assistência
Toxicológica e Toxicologistas Clínico (2023). Sistema Brasileiro de Dados de Intoxicações dos Centros de Informação e
Assistência Toxicológica.

[9] Severino FB, Vivanco P, Mix A (2022). Loxoscelismo: revisión de la literatura a propósito de un
caso. ARS Me.

[10] Caldas E﻿P (2014). Utilização racional de soros antivenenos e aprovação de protocolos
clínicos para acidentes por aranhas dos gêneros phoneutria e loxosceles, e
serpentes da familia elapidae.

[11] Rees R, Campbell D, Rieger E, King LE (1987). The diagnosis and treatment of brown recluse spider
bit. Ann Emerg Med.

[12] Wright SW, Wrenn KD, Murray L, Seger D (1997). Clinical presentation and outcome of brown recluse spider
bi. Ann Emerg Med.

[13] World Health Organization (2007). Rabies and envenomings: A neglected Public Health Issue: Report of a
Consultative Meeting, World Health Organization, Geneva, 10 January
2007.

[14] Brasil (2010). Portaria nº 2.472, de 31 de agosto de 2010. Define as terminologias
adotadas em legislação nacional, conforme disposto no Regulamento Sanitário
Internacional 2005 (RSI 2005), a relação de doenças, agravos e eventos em
saúde pública de notificação compulsória em todo o território nacional e
estabelecer fluxo, critérios, responsabilidades e atribuições aos
profissionais e serviços de saúde.

[15] Organización Panamericana de la Salud (2017). Consulta técnica sobre accidentes con animales ponzoñozos en
Latinoamérica.

[16] Braga JRM, Souza MMC, Melo IMLA, Faria LEM, Jorge RJB (2021). Epidemiology of accidents involving venomous animals in the state
of Ceará, Brazil (2007-2019). Rev Soc Bras Med Trop.

[17] Fiszon JT, Bochner R (2008). Subnotificação de acidentes por animais peçonhentos registrados
pelo sinan no estado do Rio de Janeiro no período de 2001 a
2005. Rev Bras Epidemiol.

[18] Fan HW, Vigilato MAN, Pompei JCA, Gutiérrez JM, Red de Laboratorios Públicos Productores de Antivenenos de América
Latina (2019). Situación de los laboratorios públicos productores de antivenenos
en América Latina. Rev Panam Salud Publica.

[19] Azevedo R, Azevedo FR, Ramalho RD, Goldoni PAM, Brescovit AD (2017). Acidentes causados por aranhas e escorpiões no estado do Ceará,
nordeste do Brasil: casos subnotificados e superestimados baseados na
distribuição geográfica das espécies. Pesqui Ensino Cienc Exatas Nat.

[20] Cordeiro EC, Almeida JS, Silva TS (2021). Perfil epidemiológico de acidentes com animais peçonhentos no
estado do Maranhão. Rev Cienc Plur.

[21] Wen FH, Cardoso JLC, Málaque CMS, França FOS, Sant’Anna SS, Fernandes W (2002). Influência das alterações ambientais na epidemiologia dos
acidentes ofídicos e na distribuição geográfica das serpentes de importância
médica nos estados de São Paulo e Paraná, 1988-1997. Inf Epidemiol SUS.

[22] Bochner R, Struchiner CJ (2002). Acidentes por animais peçonhentos e Sistemas Nacionais de
Informação. Cad Saude Pública.

[23] Machado C (2016). Um panorama dos acidentes por animais peçonhentos no
Brasil. Journal Health NPEPS.

[24] Pan American Health Organization (2016). Resolution CEI 158.R8 - Plan of action for the elimination of neglected
infectious diseases and post-elimination actions 2016-2022.

[25] Instituto Brasileiro de Geografia e Estatística (2022). Pernambuco: panorama.

[26] Piccolo DM (2018). Qualidade de dados dos sistemas de informação do Datasus: análise
crítica da literatura. Ci Inf Rev.

[27] Valverde JL (2003). Aspectos clínicos y epidemiológicos del loxocelismo, Hospital
Regional Docente de Trujillo, enero 2001 a noviembre 2003. Folia Dermatol.

[28] Monaco LM, Meireles FC, Abdullatif MT (2017). Animais venenosos: serpentes, anfíbios, aranhas, escorpiões, insetos e
lacraias.

[29] Schenone H, Saavedra T, Rojas A, Villarroel F (1989). Loxoscelismo en Chile: estudios epidemiológicos, clínicos y
experimentales. Rev Inst Med Trop.

